# Supplementary material for: Record-breaking emergence of upstream-downstream zonal-consistent variation in the Eurasian jet axis
Source: Nat Commun. 2026 Feb 4;17:2671. doi: 10.1038/s41467-026-68772-y (PMC13009225; doi:10.1038/s41467-026-68772-y)
Supplement: Supplementary file 1 — Supplementary Information [file 41467_2026_68772_MOESM1_ESM.pdf]

Supplementary Information for

Record-breaking emergence of upstream-downstream zonal-consistent variation in the Eurasian jet axis

This PDF file includes:

Supplementary Text S1 to S4

Supplementary Figure S1 to S21

Supplementary Table S1 to S5

Supplementary References 1 to 8

Supplementary Text 1 | Seasonality of UDZC

We have checked the UDCZ signal in other seasons, and the strong UDZC state is only found during high summer of P2.

We present the sliding correlation between the EJA and WJA indices in other seasons (DJF, MAM, SON). As shown in Supplementary Figure 2, the sliding correlation in winter or autumn is non-significant during 1940–2022. There is barely significant UDZC at $\alpha = 0.05$ at spring around 1950 and 1980 but much weaker compared to high summer and non-significant during the recent decades.

The emergence of a strong UDZC may be related to the climatological state of the jet stream. In high summer, the subtropical jet stream is at its weakest and northernmost position, making it more susceptible to signals from mid-high latitudes and corresponding to a larger Rossby wave train wavelength[1,2].

Supplementary Text 2 | Roles of ENSO, NAO, AMO, and IPO

Previous studies reveals that the Silk Road Pattern / Circumglobal pattern is modulated by ENSO[3,4] and NAO[1,5]. To investigate the role of ENSO and NAO, we firstly defined a CGSR index based on the V200 results in **Fig. 5a**. The wave train corresponding to this index is consistent with that shown in **Fig.5a**, with stronger amplitude and more pronounced propagation path (Supplementary Figure 17a, b). The correlation coefficient between the CGSR index and the projected ESWJ index reaches 0.75 ($p = 1.2\times10^{-5}$; Supplementary Figure 17e), indicating a strong relationship between the CGSR and strong ESWJ. This signal does not show a significant

linear relationship with either NAO or tropical precipitation forcing (Supplementary Figure 17c, d).

We further investigated the relationship between the projected ESWJ index and CGSR with ENSO, and found that neither concurrent ENSO nor preceding winter ENSO shows a significant relationship with ESWJ and CGSR (Supplementary Table S4).

From the aspect of decadal variability, although the reported strong UDZC events mainly occurred after the late 1990s, which coincides with the phase transition times of the IPO and AMO (Supplementary Figure 18a), the interdecadal evolution of UDZC state (21-year sliding correlation between WJA and EJA indices), shows a significant but relatively weak relationship with the AMO index (R = 0.29, $p = 3.6\times10^{-4}$, and non-significant relationship with IPO index (R = -0.10, $p = 0.22$). However, from the aspect of evolution tendency, it can be observed that the rapid increase in the UDZC since 1979 also coincides with the positive trend of the AMO and the negative trend of the IPO (Supplementary Figure 18b). This suggests that the different trends of AMO and IPO may significantly modulate the UDZC of the Eurasian jet stream.

Supplementary Text 3 | Robustness of Eurasian jet "intensity change" mode with its projected ESWJ index

The EOF mode may strongly depend on the period used. To explore such uncertainty, we conducted a number of sensitivity tests to investigate the changing EOF modes due to changing definition period of P2 (Supplementary Figure 7 and 8).

First, we choose two sub-periods of the original P2 (1999-2022): 1999-2016 and 2003-2016. For the period 1999-2016, the EOF1 still exhibit a structure highly identical to the EOF1 during 1999-2022, although with slightly weaker correlation at the low-latitudes (Supplementary

Figure 7c). Similar results are also found for the periods 2003-2016, but the structure is not very well organized, possible due to the insufficient samples (only 14 years) to extract ideal modes (Supplementary Figure 7e, f).

We also extend the period to explore the dominant modes in a longer period (Supplementary Figure 8). For three tests (1993-2022, 1987-2022 and 1981-2022), the EOF1 modes actually represents the meridional shift of Eurasian jet. However, we found that this “intensity change” mode already exists as the EOF2 (Supplementary Figure 8a, c, e), due to the relatively smaller explained variance. For the period 1999-2022, the corresponding explained variance has increased to 26.40% during 1999-2022 and become the first leading mode (Supplementary Figure 7a). This confirms that the “intensity change” mode dominant the Eurasian jet variability only after the late-1990s.

In all sensitivity tests, the spatial pattens of such “intensity change” mode is quite stable and highly consistent; and all the corresponding projected ESWJ index is all highly correlated ($R > 0.96$; Supplementary Table 2). Therefore, the “intensity change” mode is stable and robust, as well as the projected ESWJ index.

Supplementary Text 4 | Dynamical Mechanism Linking UDZC, TPH, and WNPSH

To elucidate the physical processes linking the Eurasian jet UDZC with the coupling of the TPH and WNPSH, we performed a composite analysis of vertical circulation anomalies during the P2 period (Supplementary Figure 11). During high summer, East Asia is situated at the right exit region of the eastern part of ESWJ (also called East Asian jet). The region of enhanced

westerly winds corresponds to significant upward motion, whereas the lower-latitude region dominated by easterly anomalies exhibits distinct subsidence (Supplementary Figure 11a and b).

Two primary dynamical mechanisms govern the associated secondary circulation. First, ageostrophic winds at the jet exit region typically induce upper-level convergence and subsequent subsidence on the equatorward side[6-7] (the right exit region; Supplementary Figure 11c). Second, the region south of the jet axis is characterized by negative vorticity advection (Supplementary Figure 11d), favoring geopotential height rises and subsidence (Supplementary Figure 11a).

However, these dynamical factors interact with the distinct topographic effects of the Tibetan Plateau. An intensified jet enhances this warm advection from the Tibetan Plateau[8], promoting downstream convection, precipitation, and low-level southerly convergence.

Consequently, a unique, closed secondary circulation is established south of the East Asian jet axis: (1) The strengthened jet intensifies the rainband, leading to strong rising motion and latent heat release. (2) This diabatic heating generates strong upper-level divergence (Supplementary Figure 11c). (3) Combined with the negative vorticity advection at the jet exit, this divergence sustains a robust high-pressure anomaly in the upper troposphere (the eastward extended TPH). (4) Driven by the ageostrophic flow, air converges and sinks at lower latitudes, extending the high-pressure system downward to form a significant low-level anticyclonic anomaly (the westward extended WNPSH). (5) This low-level anticyclone suppresses local convection while enhancing the northward transport of moisture, which further fuels the rainband precipitation, thereby closing the positive feedback loop (Supplementary Figure 12).

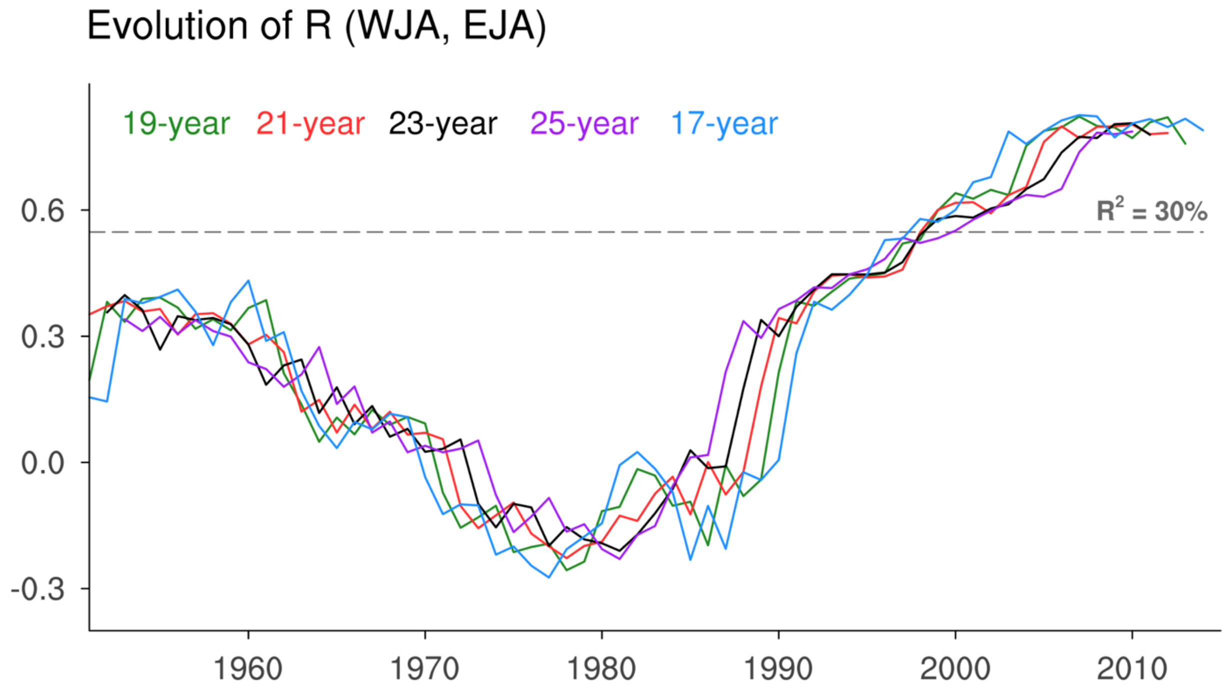

Supplementary Figure 1 | Evolution of High Summer UDZC in different sliding window. Sliding correlation of WJA and EJA indices in 17-year (blue), 19-year (green), 21-year (red), 23-year (black) and 25-year (purple) sliding window using ERA-5.

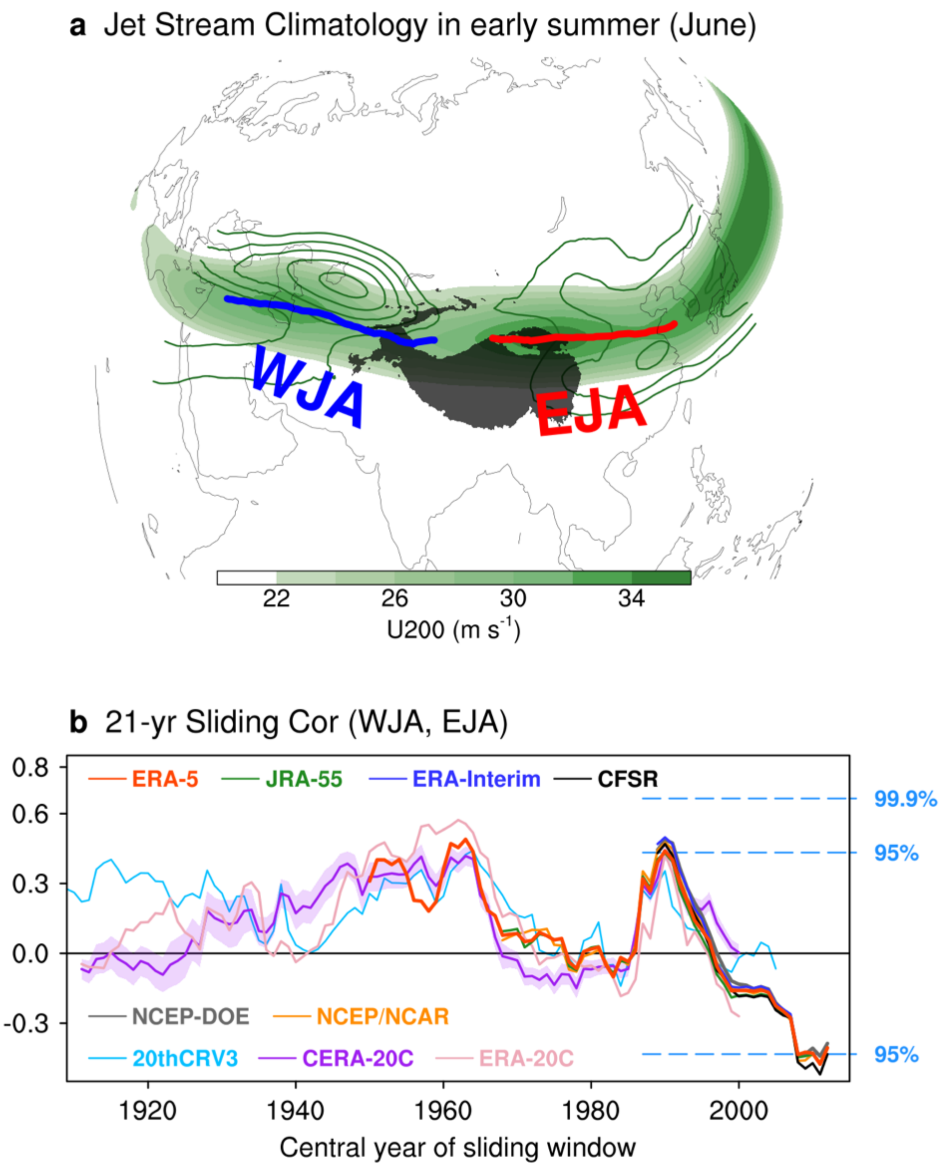

Supplementary Figure 2 | Change in UDZC of Eurasian jet in early summer. a–b Same as Fig. 1a, c, except for June. The blue dash lines indicate the 95% and 99% confidence level based on the student's *t*-test.

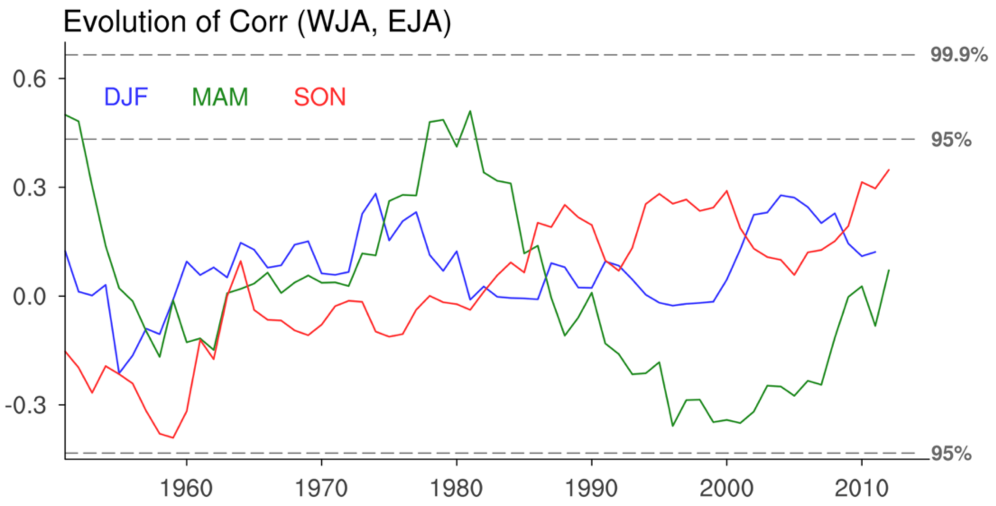

Supplementary Figure 3 | Seasonality of UDZC interdecadal evolution. The 21-year sliding correlation between WJA and EJA indices during winter (DJF, blue), spring (MAM, green) and autumn (SON, red), using results from ERA-5.

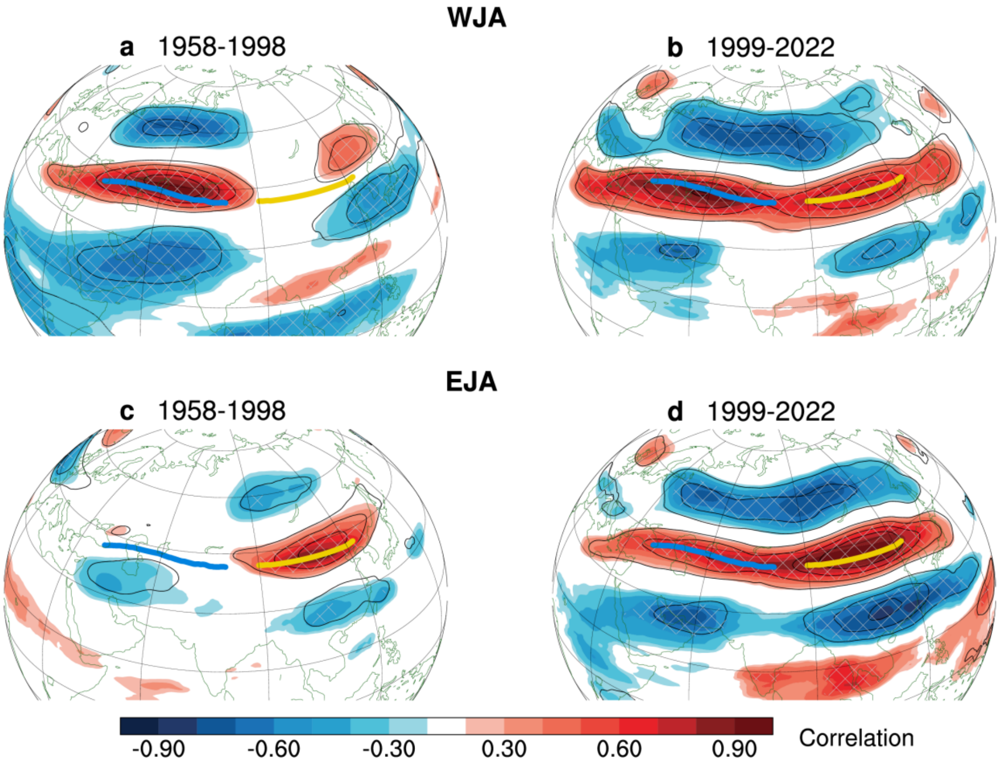

Supplementary Figure 4 | Enhanced relationship between WJA and EJA. a–b WJA related U200 anomalies (shading: correlation map; contour: regression map, units: m s^{-1}) at P1(**a**) and P2 (**b**), respectively, with statistically significant values after controlling for the false discovery rate ($\alpha_{FDR} = 0.1$) hatched. **c–d** Same as **a–b**, but for the EJA index. The blue and yellow lines indicate the WJA and EJA, respectively.

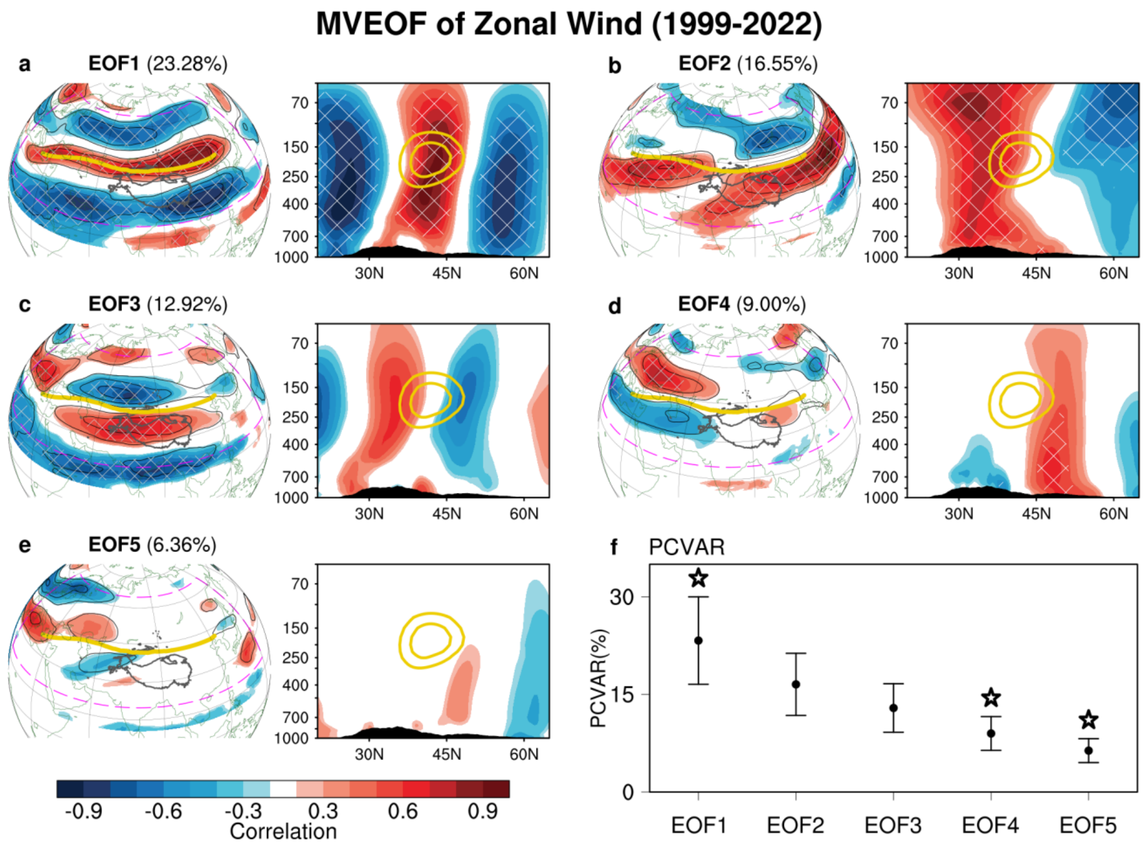

Supplementary Figure 5 | The first five leading EOF modes of Eurasian jet during P2. The EOF is performed on the combination of U200 (20°–65°N; 0°–150°E) and zonally averaged zonal wind (20°–65°N; 0°–150°E; 500 hPa–100 hPa). **a** The EOF1 mode of U200 anomalies (left; shading: correlation map; contour: regression map, units: m s^{-1}) and zonally averaged vertical section (right) during P1. Hatching indicates statistically significant values after controlling for the false discovery rate (α_{FDR} = 0.10). The yellow line in left indicates the climatology of jet stream axis and yellow contours in right represent the climatology of Eurasian jet at 20 and 25 m s^{-1}. **b–e** Same as a, but for the EOF2–5 mode.
Box with purple dash line denotes the regions for performing EOF. **f** Percentage of explained variance for each mode. Error bar denotes the lower and upper test bounds according to North's rule. The error bar with star above denotes this mode is significantly separated to other modes.

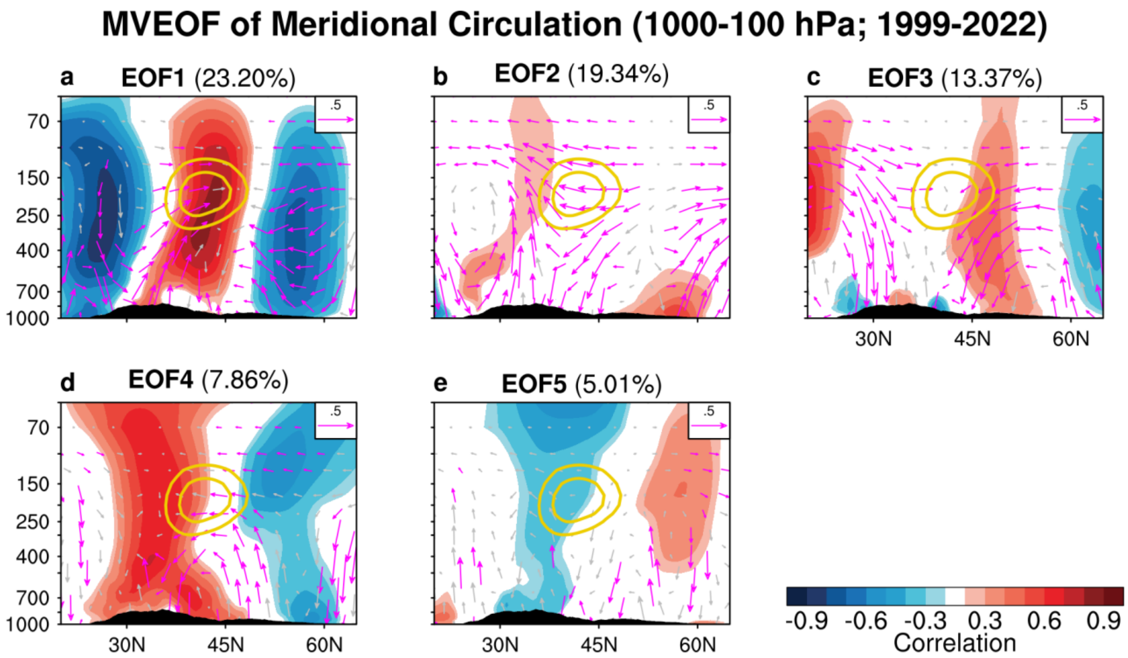

Supplementary Figure 6 | The first five leading MVEOF modes of meridional circulation (1000–100 hPa) during P2. a–e Shadings represents the correlation filed of zonal wind with PCs with vectors representing the regressed vertical circulation against normalized PCs, with 95% confidence colored. Yellow contours represent the climatology of Eurasian jet at 20 and 25 m s^{-1}.

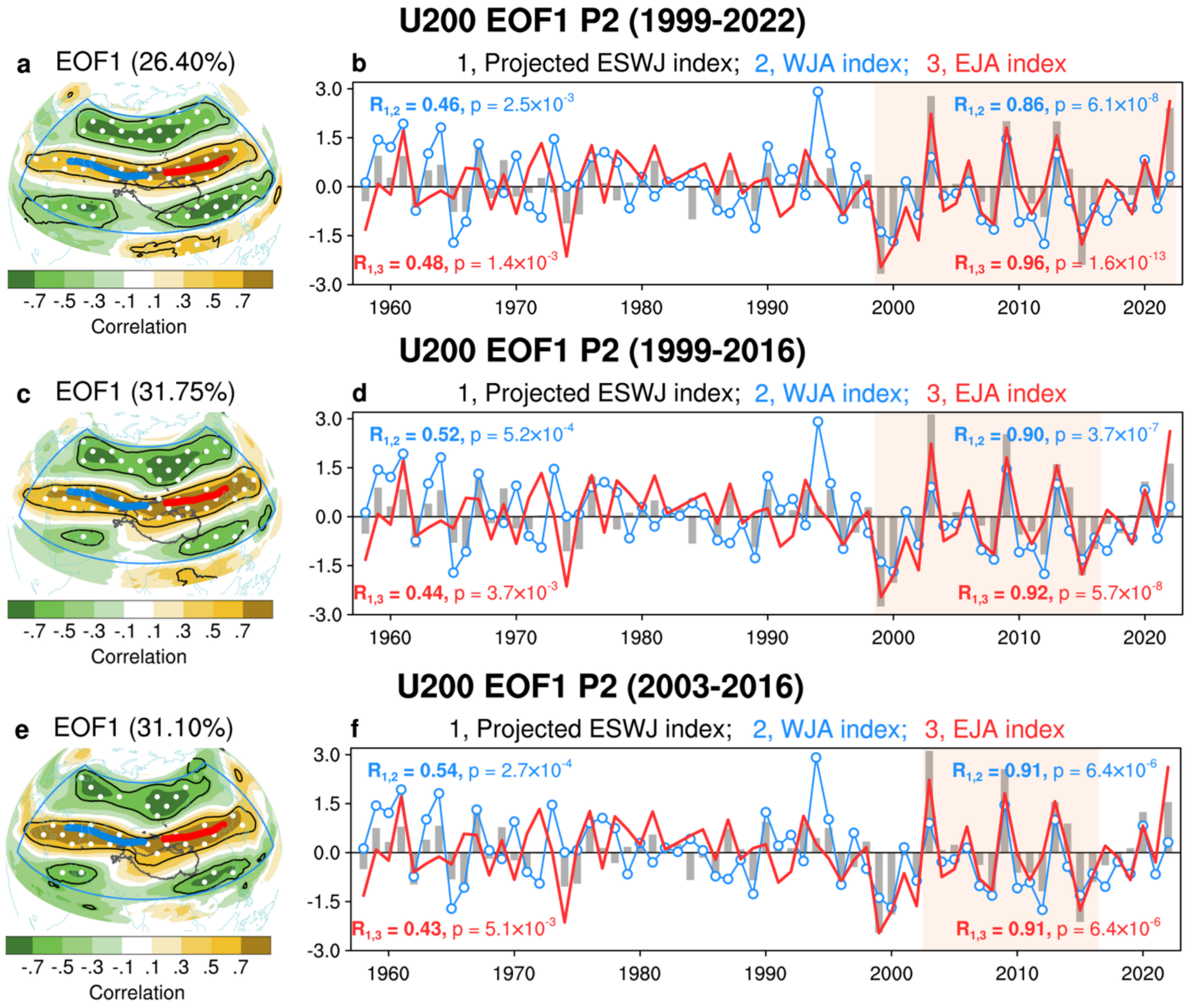

Supplementary Figure 7 | Sensitivity tests of EOF mode that can reflect the UDZC phenomenon and corresponding projected ESWJ index to the period-choice of P2: Shorten the selected time period of P2.

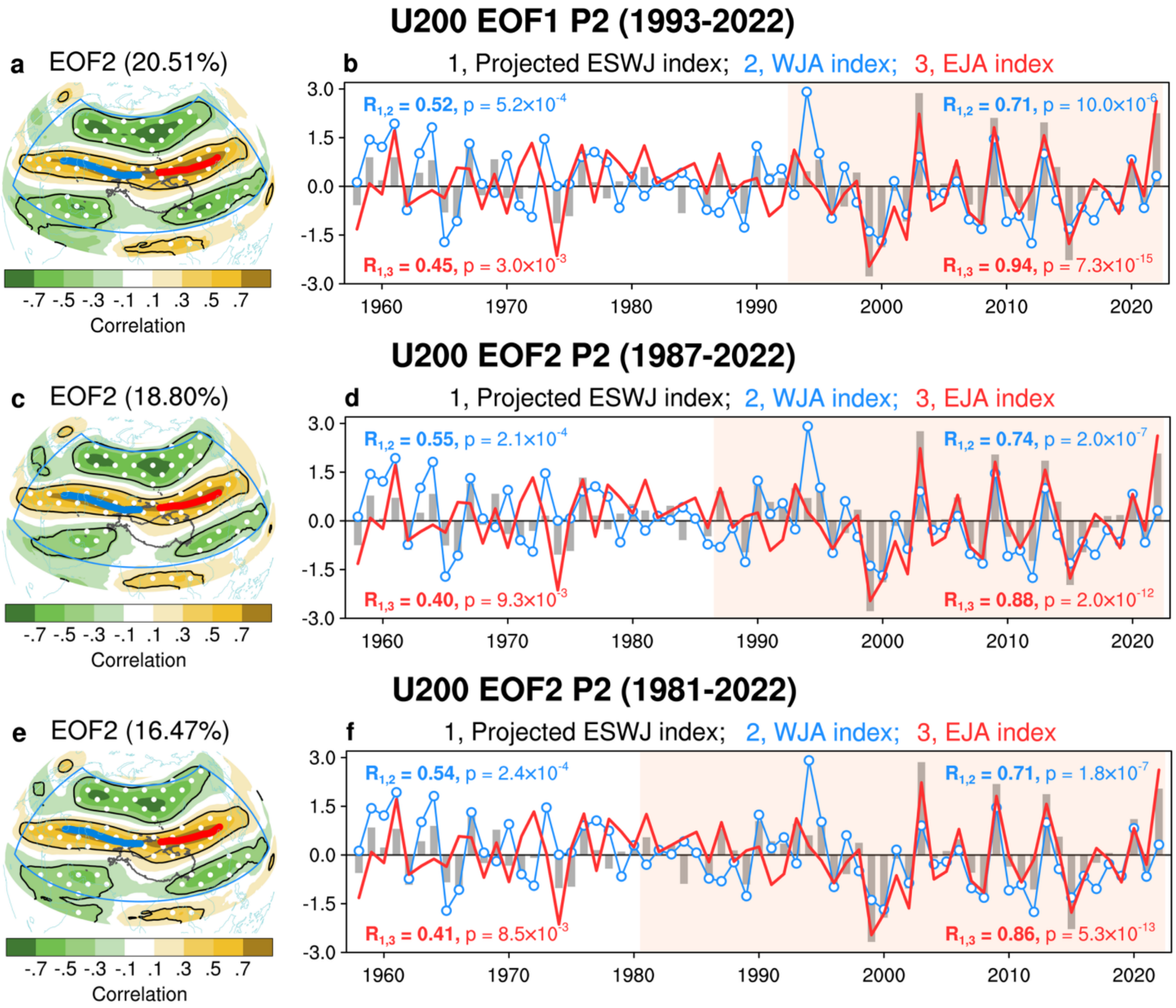

Supplementary Figure 8 | Sensitivity tests of EOF mode that can reflect the UDZC phenomenon and corresponding projected ESWJ index to the period-choice of P2: Lengthen the selected time period of P2.

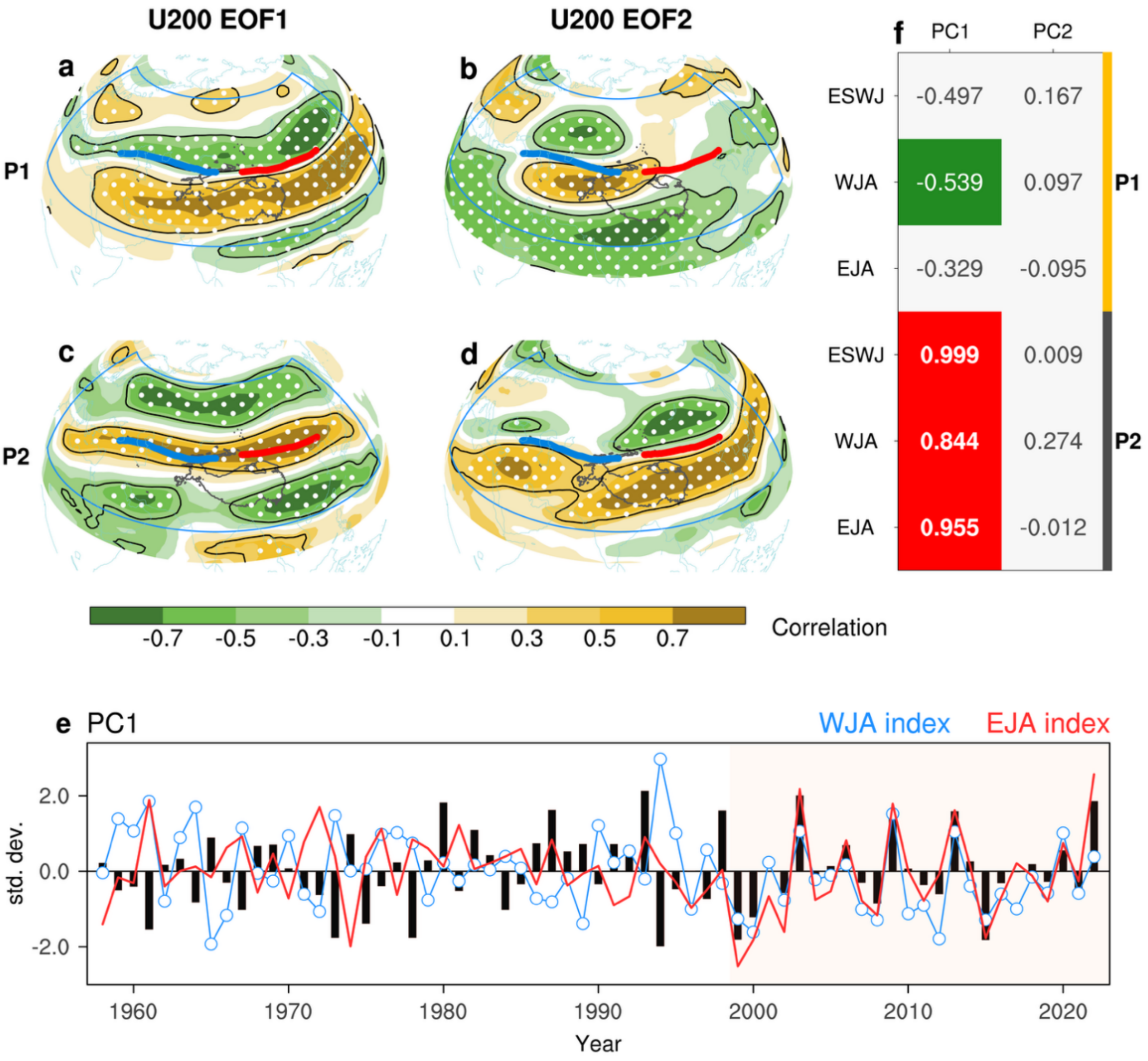

Supplementary Figure 9 | Shifting Eurasian westerly modes. Same as **Fig. 2**, but produced using JRA-55

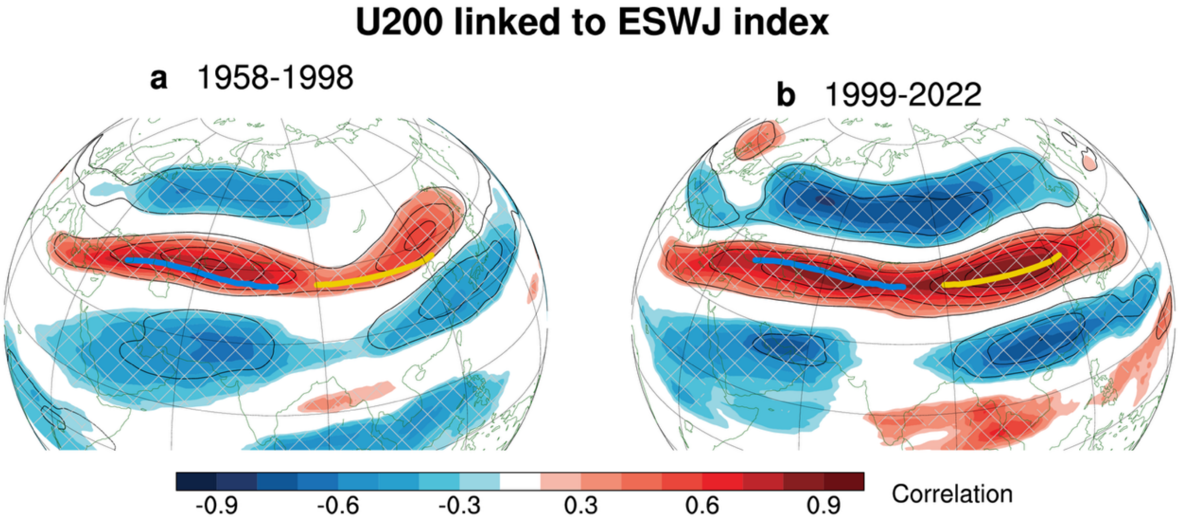

Supplementary Figure 10 | Different spatial pattern of projected ESWJ index during P1 and P2. a–b ESWJ related U200 anomalies (shading: correlation map; contour: regression map, units: m s^{-1}) at P1(a) and P2 (b), respectively, with statistically significant values after controlling for the false discovery rate ($\alpha_{FDR} = 0.1$) hatched.

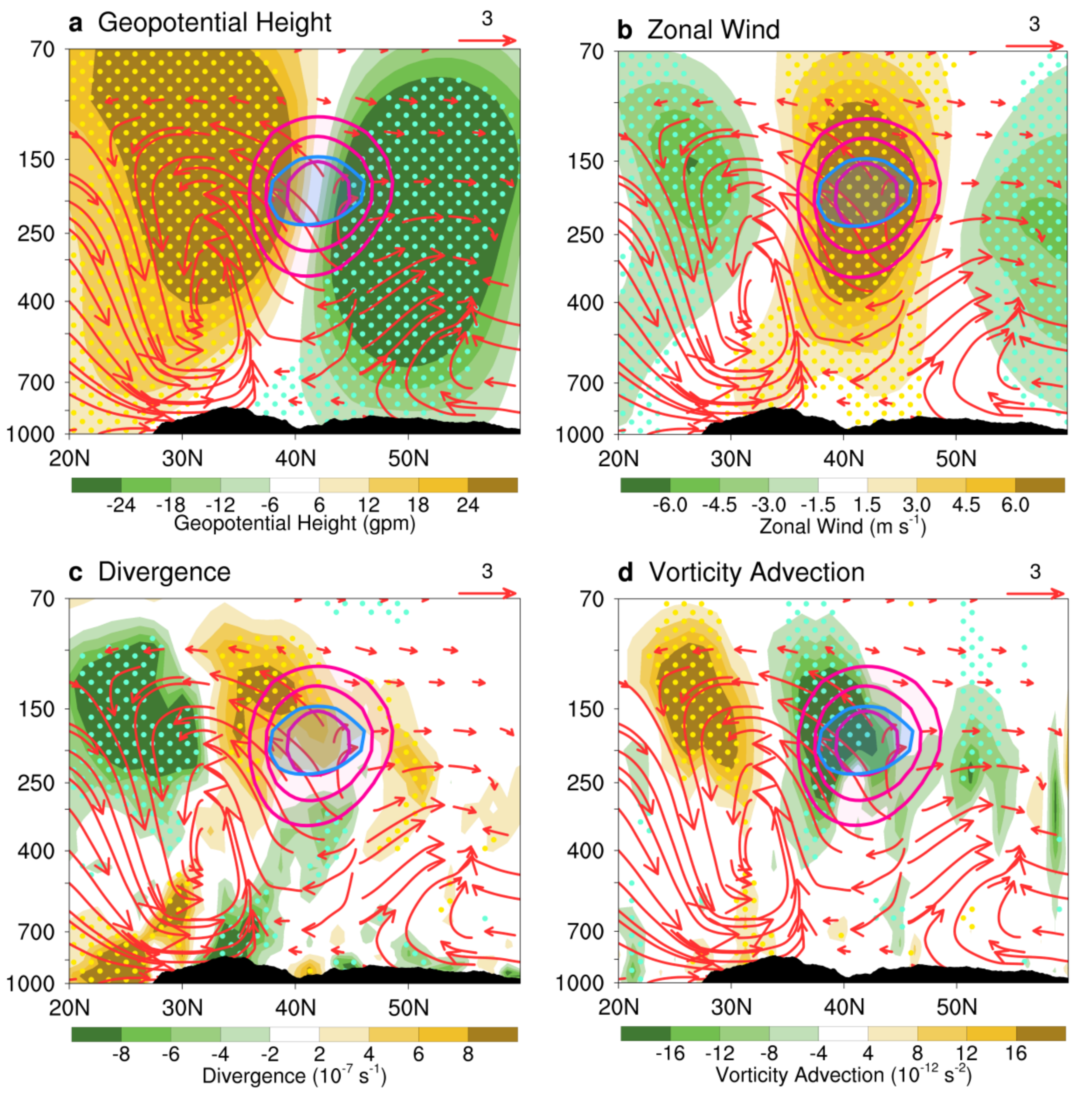

Supplementary Figure 11 | Meridional section of secondary circulation linking strengthened Eurasian jet to enhanced subtropical highs. a Composite difference of geopotential height anomalies (zonal mean from 90°N–135°N; shading; units: gpm) against the projected ESWJ jet index during P2, with 95% confidence stippled. Red vectors representing the composite difference vertical circulation against normalized PCs (meridional wind and vertical velocity). Deep pink (blue) contours represent the composite mean of Eurasian jet during strong (weak) jet year at 20 and 25 m s^{-1}.

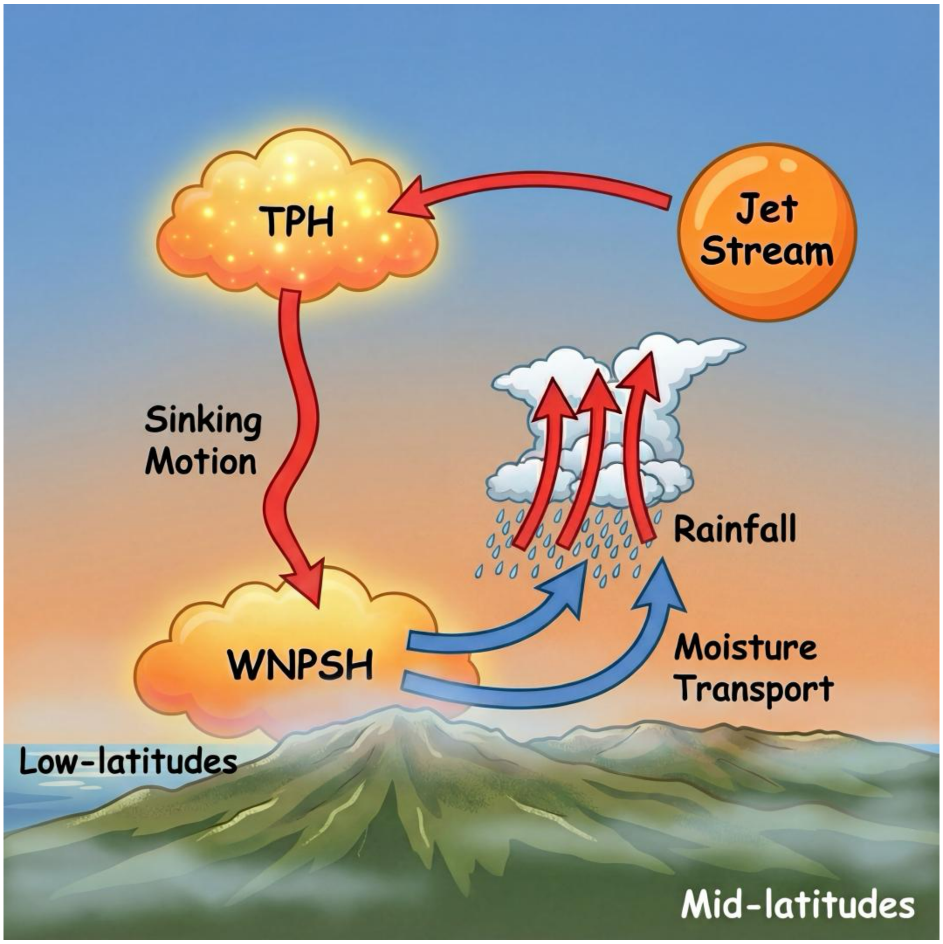

Supplementary Figure 12 | Schematic diagram of secondary circulation at East Asian landmass related to strengthened Eurasian jet at the south flank of Eurasian jet.

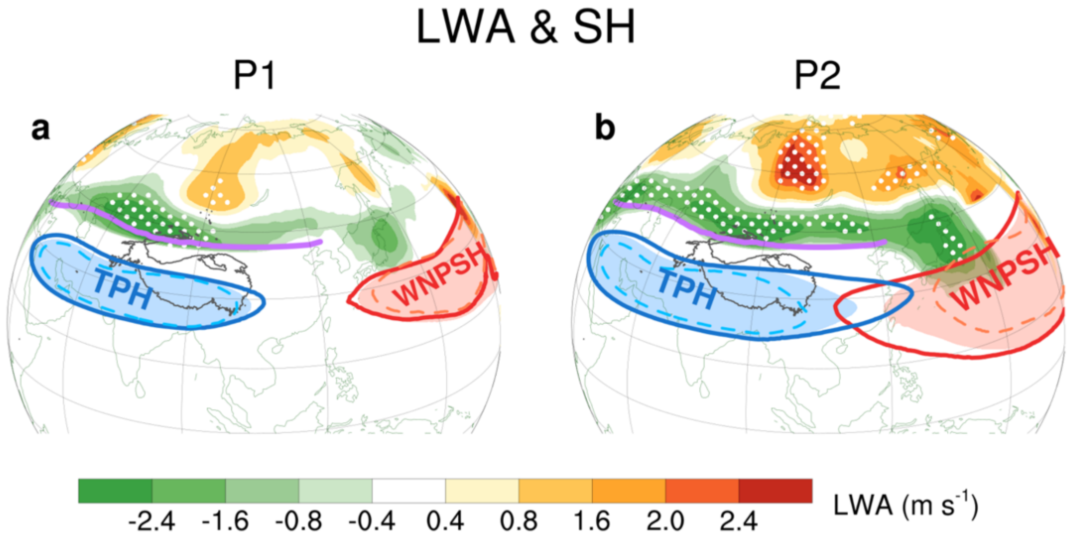

Supplementary Figure 13 | Regime shifts linked to enhancing UDZC of the Eurasian jet axis. a–b Same as **Fig. 4a** and **c**, but the strong (weak) jet years for calculating composite TPH and WNPSH is selected based on the threshold of 1 std. dev..

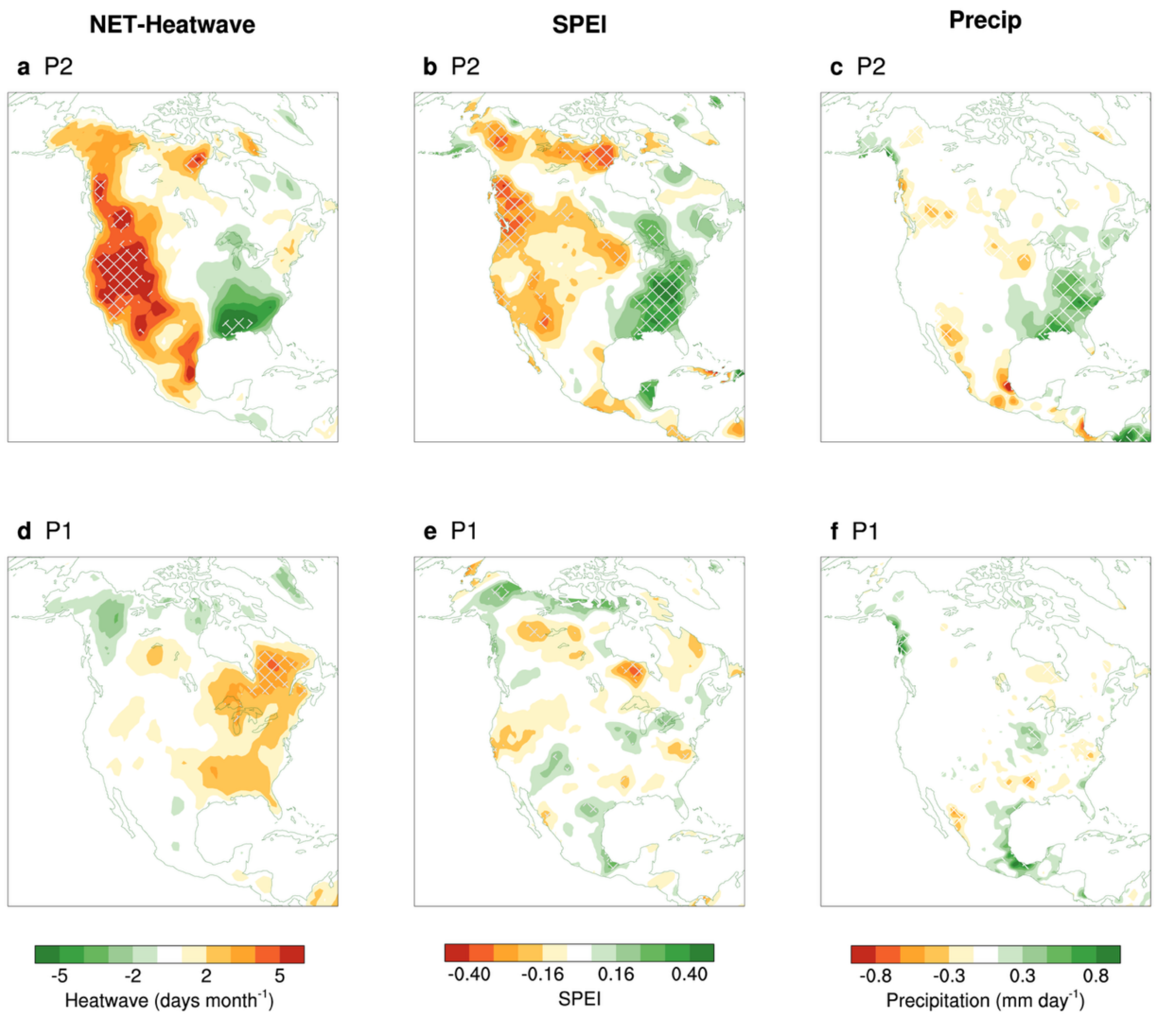

Supplementary Figure 14 | Enhanced impact of UDZC on North America weather extremes and climate. a Regression map of NET-heatwave frequency (units: days month^{-1}) against the projected ESWJ index during P2, with 95% confidence hatched. **b** Similar to (**a**), but for the SPEI anomalies. Note that the white dots in (**a**) and (**b**) mark the same regions with significant anomalies of compound extreme hot and dry events. **c** Similar to (**a**), but for the precipitation anomalies (units: mm day^{-1}). **d–f** Similar to (**a–c**), but for the P1.

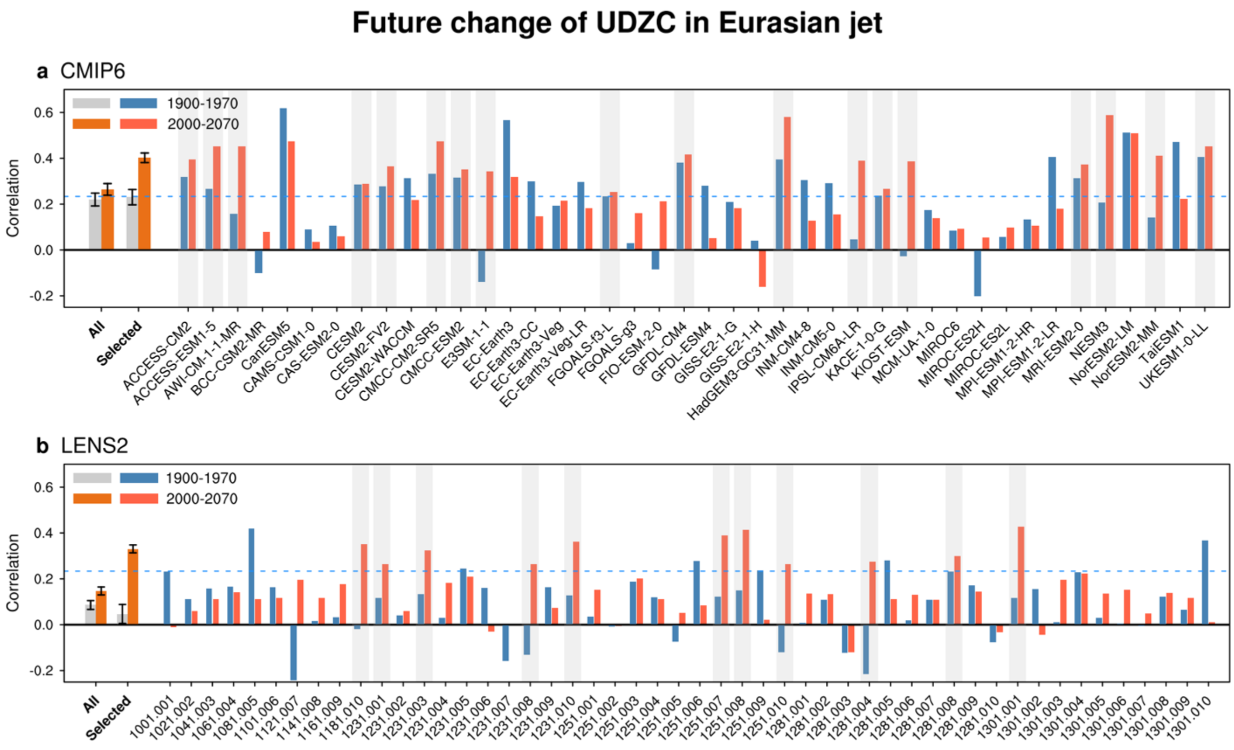

Supplementary Figure 15 | Future change of UDZC in Eurasian jet in high summer. a Comparison of the Correlation coefficient between WJA and EJA indices in the history (1900–1970; blue bars) and future (2000–2070; orange bars) climate in 42 CMIP6 models. Models (members) simulate a significant increase in UDZC of Eurasian jet are shows in grey background color. The multi-model means of selected models, unselected models and all models are also shown in grey (history) and orange (future) bar. **b** Same as **a**, but obtained from 50 members of CESM2 Large Ensemble (LENS2). Error bars in the multi-model mean (ensemble mean) are SD of 10000 realizations (see “Bootstrap test” in Methods).

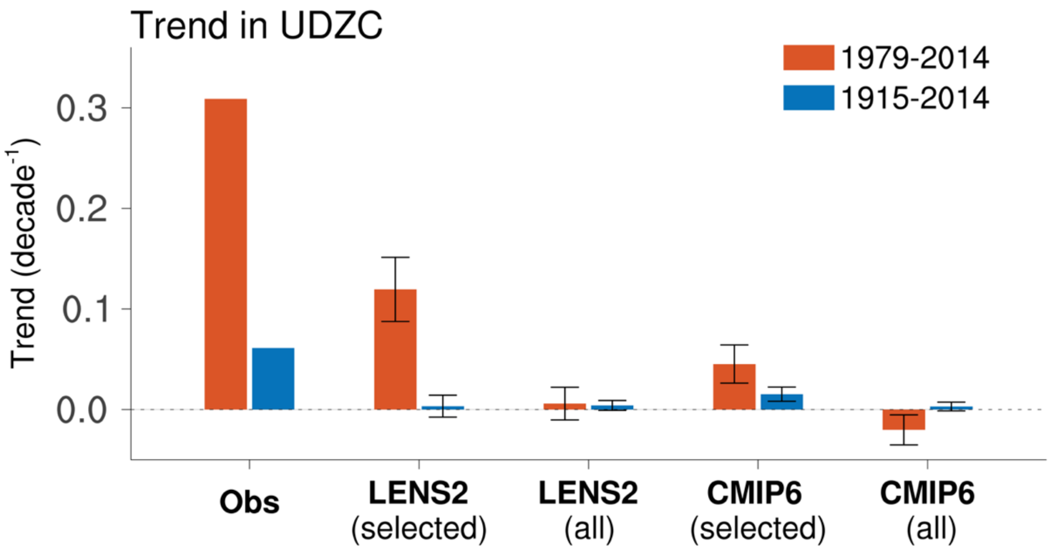

Supplementary Figure 16 | Trend of UDZC in observation and simulation. Trend of UDZC in observation and simulation during satellite era (1979–2014; orange bars) and past century (1915–2014; blue bars). Error bars represent the corresponding std. dev. of 10000 realizations (see “Bootstrap test” in Methods).

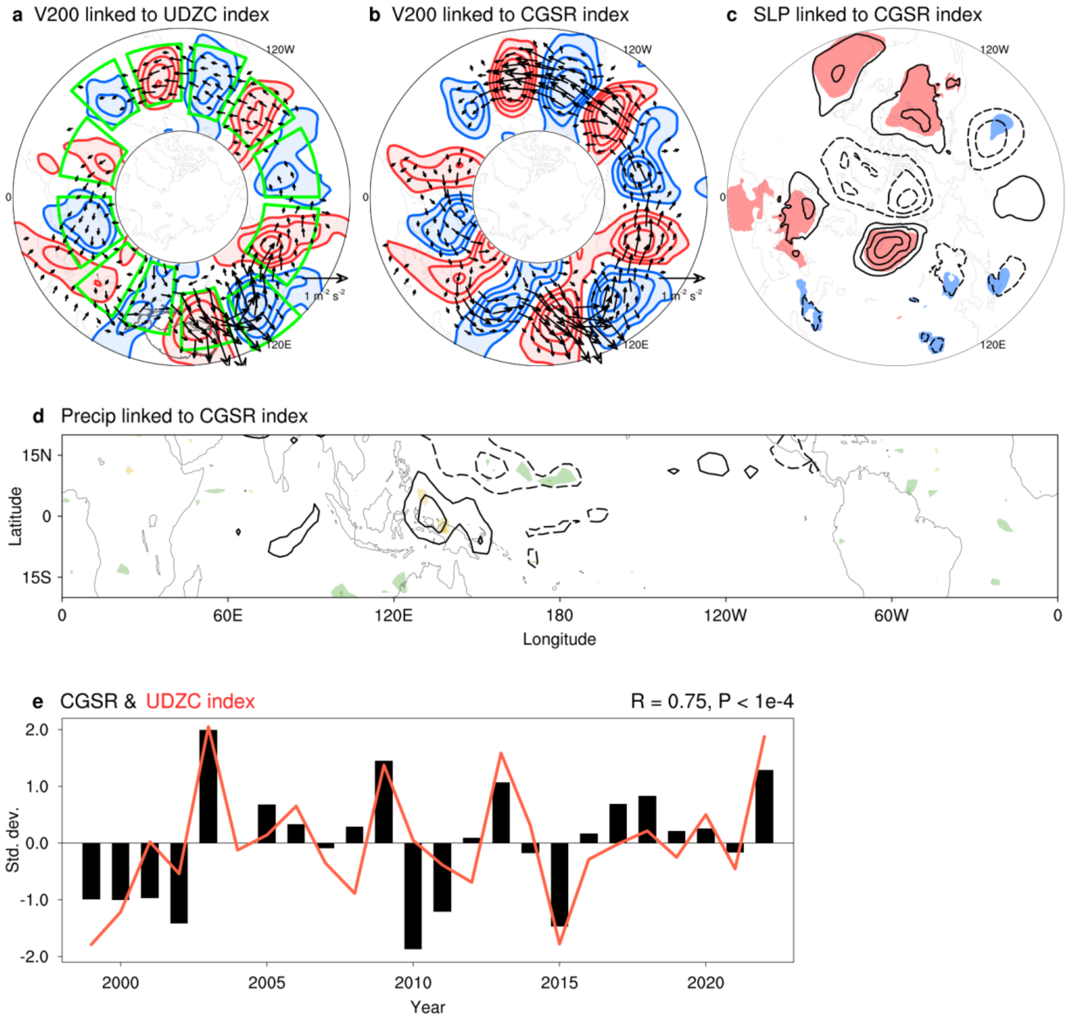

Supplementary Figure 17 | CGSR index and related atmospheric anomalies. a–b, regression map of V200 (unit: m s^{-1}) against the normalized projected ESWJ index (**a**) and CGSR index (**b**), with vectors represent regressed WAF (unit: m^{-2} s^{-2}). Green boxes in (**a**) outlines the definition regions to defining CGSR index. **c–d**, regression maps of SLP (**c**; unit: Pa; contour interval: 40 Pa) and precipitation (**d**; units: mm day^{-1}) against standardized CGSR index, with shadings represents 95% confidence. **e**, year-to-year variation of standardized CGSR index (black bar) with the normalized projected ESWJ index (red curve).

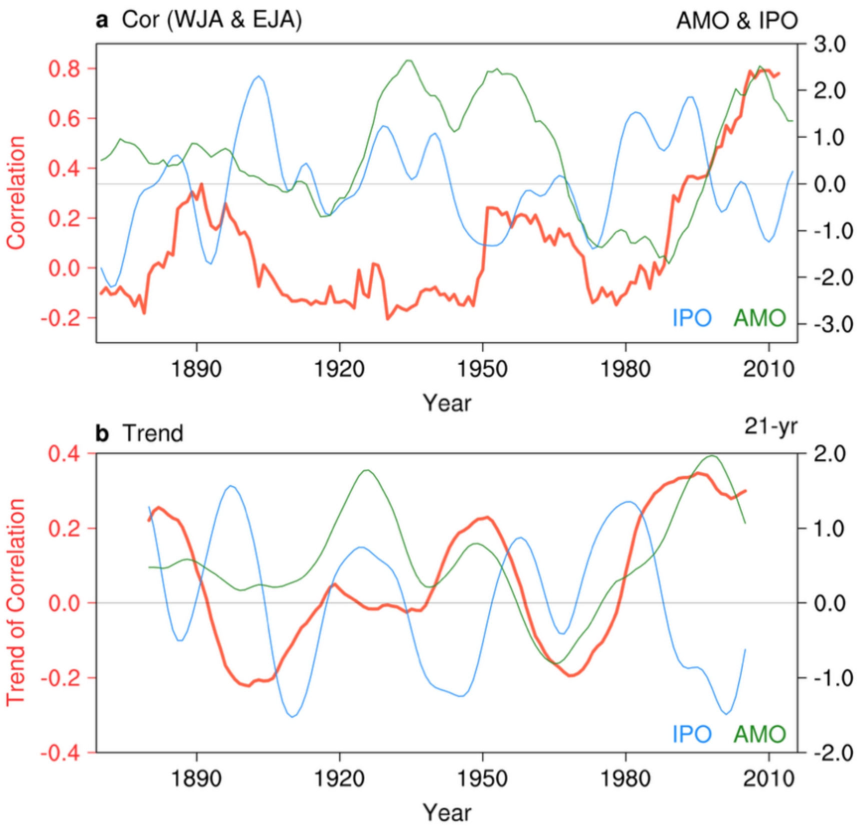

Supplementary Figure 18 | Modulation of AMO and IPO on UDZC. a, The 21-year sliding correlation of WJA and EJA indices (red curve), with AMO (green) and IPO (blue) index. **b**, similar to a, but their 21-yr sliding trend.

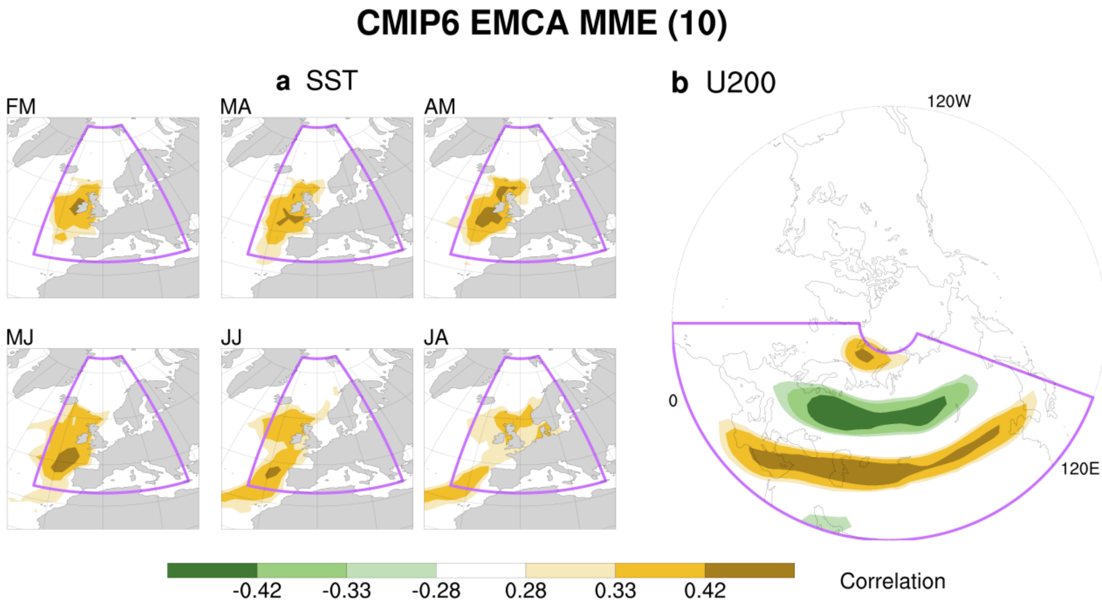

Supplementary Figure 19 | CMIP6 model mean of EMCA1 mode of co-varying North Atlantic SST evolution and Eurasian circulation (U200) in high summer. a–b, multi model mean of the homogeneous correlation maps against the temporal coefficients corresponding to EMCA1, with 90% confidence colored. The blue line in (b) indicates the Eurasian jet axis. Purple boxes denote the regions for EMCA1. Those selected 10 models include ACCESS-ESM1-5, AWI-CM-1-1-MR, CAMS-CSM1-0, CESM2, CESM2-WACCM, INM-CM5-0, IPSL-CM6A-LR, MIROC-ES2H, NorESM2-MM and UKESM1-0-LL.

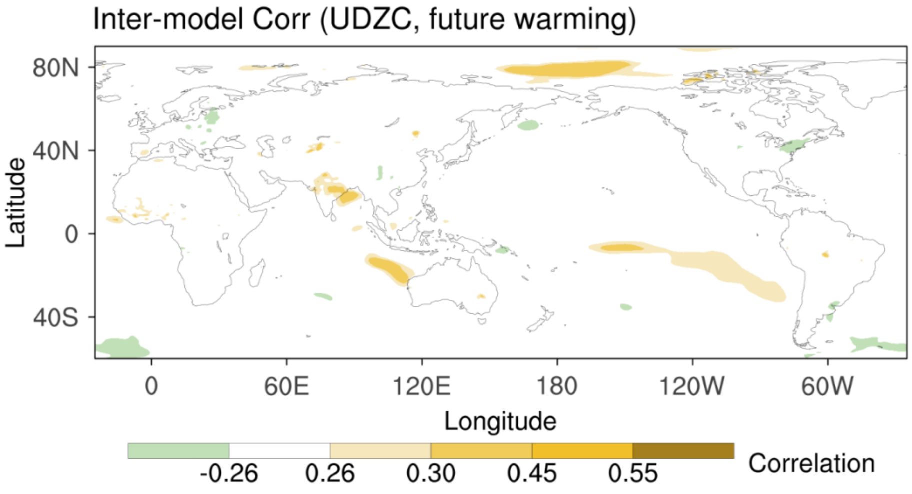

Supplementary Figure 20 | Inter-member correlation map of future surface warming amplitude (2000–2070 minus 1900–1970) with future UDZC of Eurasian jet. Similar to Fig. 7b, but using output form LENS2 Project.

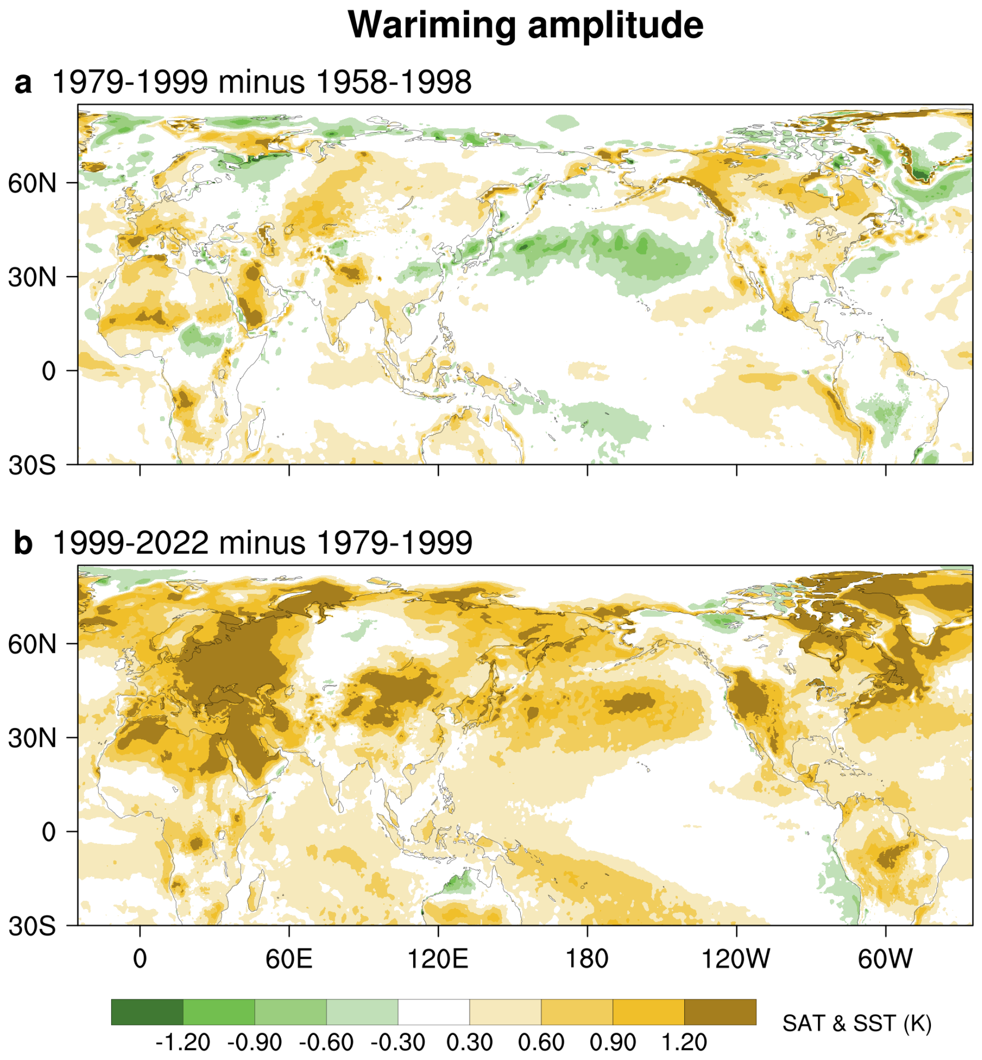

Supplementary Figure 21 | Amplitude of warming observed among different periods. a–b Difference of warming in surface air temperature and sea surface air temperature (units: K) between 1979–1999 and 1959–1998 (**a**) and between 1999–2022 and 1979–1999 (**b**).

Supplementary Table 1 | A list of sensitivity test on the domain selection of WJA and EJA. The corresponding correlation coefficient during P1 (R1) and P2 (R2) are shown. The R with $p < 0.05$ is marked with bold and red color.

	WJA domain	EJA domain	R1	R2
**Test 1**	40°–80°E	90°–130°E	0.09	**0.79**
**Test 2**	40°–85°E	90°–130°E	0.11	**0.82**
**Test 3**	40°–80°E	90°–135°E	0.13	**0.78**
**Test 4**	40°–85°E	90°–135°E	0.15	**0.82**
**Test 5**	40°–85°E	90°–140°E	0.17	**0.82**
**Test 6**	35°–85°E	90°–140°E	0.18	**0.81**

Supplementary Table 2 | Sensitivity test of projected ESWJ indices. Note that here all the correlations (R) are calculated for the projected ESWJ indices during 1958–2022, but obtained from different projection reference-period (as shown in Table). In other words, the periods listed in this Table indicate the projection reference-period for the EOF mode that can reflect the UDZC phenomenon (see Supplementary Figs. 7 and 8). It is clear to see that all the correlations exceed 0.96 ($p < 0.001$), suggesting that the projected ESWJ index is not sensitive to the projection reference-period.

EOF mode and reference-period for the projected ESWJ	EOF2 1981-2022	EOF2 1987-2022	EOF2 1993-2022	EOF1 1999-2022	EOF1 1999-2016	EOF1 2003-2016
EOF2 1981-2022	1	0.989	0.997	0.985	0.983	0.985
EOF2 1987-2022	0.989	1	0.988	0.964	0.971	0.969
EOF2 1993-2022	0.997	0.988	1	0.992	0.980	0.979
EOF1 1999-2022	0.985	0.964	0.992	1	0.967	0.964
EOF1 1999-2016	0.983	0.971	0.980	0.967	1	0.995
EOF1 2003-2016	0.985	0.969	0.979	0.964	0.995	1

Supplementary Table 3 | Selected strong/weak Eurasian jet years in two sub-periods. Strong (weak) Eurasian jet year are selected as the year whose corresponding value of projected ESWJ index is above + 0.5 (below -0.5).

	**Strong Eurasian jet years**	**Weak Eurasian jet years**
**P1**	1959, 1961, 1963, 1964, 1967, 1969, 1972, 1976, 1981, 1987, 1990, 1991, 1993, 1995	1958, 1962, 1965, 1966, 1968, 1970, 1974, 1975, 1978, 1984, 1986, 1989, 1996, 1997
**P2**	2003, 2006, 2009, 2013, 2020, 2022	1999, 2000, 2002, 2008, 2012, 2015

Supplementary Table 4 | Correlation coefficients between different ENSO index, projected ESWJ and CGSR index.

	Nino3 (JF)	Nino4 (JF)	Nino3.4 (JF)	Nino3 (JA)	Nino4 (JA)	Nino3.4 (JA)
ESWJ index	0.04	0.22	0.13	-0.17	0.04	-0.09
CGSR index	-0.01	0.05	0.01	0.02	0.04	0.13

Supplementary Table 5 | A list CMIP6 models used with corresponding institutions and resolutions.

**No**	**Model Name**	**Institute**	**Resolution**
**1**	ACCESS-CM2	CSIRO-ARCCSS	250 km
**2**	ACCESS-ESM1-5	CSIRO	250 km
**3**	AWI-CM-1-1-MR	AWI	100 km
**4**	BCC-CSM2-MR	BCC	100 km
**5**	CAMS-CSM1-0	CAMS	100 km
**6**	CAS-ESM2-0	CAS	100 km
**7**	CanESM5	CCCma	500 km

**8**	CESM2	NCAR	100 km
**9**	CESM2-FV2	NCAR	250 km
**10**	CESM2-WACCM	NCAR	100 km
**11**	CMCC-CM2-SR5	CMCC	100 km
**12**	CMCC-ESM2	CMCC	100 km
**13**	E3SM-1-1	E3SM-Project RUBISCO	100 km
**14**	EC-Earth3	EC-Earth-Consortium	100 km
**15**	EC-Earth3-CC	EC-Earth-Consortium	100 km
**16**	EC-Earth3-Veg	EC-Earth-Consortium	100 km
**17**	EC-Earth3-Veg-LR	EC-Earth-Consortium	250 km
**18**	FGOALS-f3-L	CAS	100 km
**19**	FGOALS-g3	CAS	100 km
**20**	FIO-ESM-2-0	FIO-QLNM	100 km
**21**	GFDL-CM4	NOAA-GFDL	100 km
**22**	GFDL-ESM4	NOAA-GFDL	100 km
**23**	GISS-E2-1-G	NASA-GISS	250 km
**24**	GISS-E2-1-H	NASA-GISS	250 km
**25**	HadGEM3-GC31-MM	MOHC	100 km
**26**	INM-CM4-8	INM	100 km
**27**	INM-CM5-0	INM	100 km
**28**	IPSL-CM6A-LR	IPSL	250 km
**29**	KACE-1-0-G	NIMS-KMA	250 km
**30**	KIOST-ESM	KIOST	250 km
**31**	MCM-UA-1-0	UA	250 km
**32**	MIROC-ES2L	MIROC	500 km
**33**	MIROC-ES2H	MIROC	250 km
**34**	MIROC6	MIROC	250 km
**35**	MPI-ESM1-2-HR	MPI-M DWD DKRZ	100 km
**36**	MPI-ESM1-2-LR	MPI-M AWI DKRZ DWD	250 km
**37**	MRI-ESM2-0	MRI	100 km
**38**	NESM3	NUIST	250 km
**39**	NorESM2-LM	NCC	250 km
**40**	NorESM2-MM	NCC	100 km
**41**	TaiESM1	AS-RCEC	100 km
**42**	UKESM1-0-LL	MOHC NERC NIMS-KMA NIWA	100 km

Supplementary References:

1. Hong, X., Lu, R. & Li, S. Differences in the Silk Road Pattern and Its Relationship to the North Atlantic Oscillation between Early and Late Summers. *J. Clim*. 31, 9283–9292 (2018).
2. Zhang, Y., Kuang, X., Guo, W. & Zhou, T. Seasonal evolution of the upper-tropospheric westerly jet core over East Asia. *Geophys. Res. Lett.* **33**, 2006GL026377 (2006).
3. Yang, X., Huang, P., Liu, Y. & Chen, D. An interdecadal enhancement of the impact of ENSO on the summer Northeast Asia circulation around 1999/2000 through the Silk Road Pattern. *J. Clim.* 1–40 (2022)
4. Tang, S. et al. Recent changes in ENSO's impacts on the summertime circumglobal teleconnection and mid-latitude extremes. *Nat. Commun.* **16**, 646 (2025).
5. Hong, X., Lu, R., Chen, S. & Li, S. The relationship between the North Atlantic Oscillation and the Silk Road pattern in summer. *J. Clim.* (2022)
6. Li, X., Zheng, J., Wang, C., Lin, X. & Yao, Z. Unraveling the roles of jet streams on the unprecedented hot July in Western Europe in 2022. npj Clim. Atmos. Sci. 7, 323 (2024).
7. Shou, S., Li, S., Shou, Y. & Yao, X. Front and jet stream. *An Introduction to Mesoscale Meteorology* 73–115 (Springer, Singapore, 2023).
8. Sampe, T. & Xie, S. Large-Scale Dynamics of the Meiyu-Baiu Rainband: Environmental Forcing by the Westerly Jet. *J. Clim*. **23**, 113–134 (2010).
